# Multi-scale model of drug induced adaptive resistance of Gram-negative bacteria to polymyxin B

**DOI:** 10.1371/journal.pone.0171834

**Published:** 2017-03-23

**Authors:** Wojciech Krzyzanski, Gauri G. Rao

**Affiliations:** 1 Department of Pharmaceutical Sciences, School of Pharmacy Practice and Pharmaceutical Sciences, University at Buffalo, Buffalo, New York, United States of America; 2 Division of Pharmacotherapy and Experimental Therapeutics, UNC Eshelman School of Pharmacy, The University of North Carolina at Chapel Hill, Chapel Hill, North Carolina, United States of America; Azienda Ospedaliera Universitaria di Perugia, ITALY

## Abstract

The purpose of this report is to apply multi-scale modeling using the theory of physiologically structured populations (PSP) to develop a mathematical model for antimicrobial resistance based on a heterogeneous distribution of receptors and affinities among bacterial cells. The theory has been tested on data obtained from an *in vitro* static time-kill infection model analyzing the pharmacodynamics of polymyxin B against Gram-negative bacteria. The drug binding parameter K_D_ (dissociation equilibrium constant) is assumed to vary between the bacterial cells. The PSP model describes the time course of the density distribution of K_D_ upon exposure to cytotoxic drug concentrations. The drug increases the hazard of cell death as a function of receptor occupancy. The initial distribution of K_D_ is described by the Weibull function. Time-kill data were used for model qualification. *In vitro* static time-kill experiments to evaluate the rate and extent of killing due to polymyxin B against two *Klebsiella pneumoniae* clinical isolates with differing susceptibilities to polymyxin B were performed over 48 h. The time-kill kinetics data of bacterial load cfu (colony forming units)/mL was used for model qualification. The resistant bacterial population is determined by the balance between growth rate and hazard of cell death controlled by polymyxin B concentrations. There exists a critical K_D_ value below which cells continue to grow. Estimates of shape parameters for distributions of K_D_ yielded unimodal distributions with the modes at 0 nM and the right tails containing approximately 25% of the bacteria. Our findings support a hypothesis that resistance of *Klebsiella pneumoniae* to polymyxin B can be at least partially attributed to a drug-induced selection of a subpopulation due to heterogeneity of polymyxin B receptor binding in the bacterial population.

## Introduction

Prevalence of Gram-negative bacteria resistant to almost all currently available antibiotics is rapidly escalating at an alarming rate. This poses a serious clinical challenge as it limits our treatment options against infections due to resistant Gram-negative pathogens. Mechanisms of bacterial resistance have been classified into three categories: intrinsic, acquired, and adaptive [1]. Intrinsic resistance comprises all the inherent mechanisms that restrain the effects of antimicrobials by reducing the intracellular drug concentration (such as Gram-negative outer membrane that is impermeable to several antimicrobial classes and the increased expression of numerous efflux pumps). Acquired resistance refers to the acquisition of new genes by the incorporation of genetic material or a result of spontaneous mutations enabling bacteria to withstand higher antibiotic concentrations. Adaptive resistance is a temporary increase in the ability of bacteria to survive drug pressure with alterations to the gene and/or protein expression in response to the environmental trigger. Adaptive resistance, in contrast to intrinsic or acquired resistance, is triggered in response to the selective pressure exerted by the use of antibiotics. Bacterial phenotype can contain a high degree of molecular heterogeneity. An example of adaptive drug resistance is a drug-induced selection of a resistant bacterial subpopulation that were present in the original bacterial population [2].

Polymyxin B is a polycationic cyclic antimicrobial peptide with a molecular mass 1,200 Da carrying five positively charged residues of diaminobutyric acid. Polymyxin B binds to lipopolysaccharide (LPS) expressed on the outer membrane of Gram-negative bacteria such as *Escherichia coli* and *Klebsiella pneumoniae* by displacing divalent cations Mg^2+^ and Ca^2+^ that cross-bridge between adjacent negatively charged phosphate groups [3]. Upon biding to LPS, polymyxin B forms a complex with LPS that penetrates the outer membrane of the bacteria. The bactericidal effect of polymyxin B is attributed to its interaction with the cytoplasmic membrane that alters its functions such as active transport and respiration [4].

Gram-negative bacteria alter their LPSs to protect themselves against the bactericidal effects of polymyxin B. One of the most common modifications of LPS resulting in reduced binding affinity to polymyxin is the cationic substitution of its phosphate groups by 4-amino-4-deoxy-L-arabinose (L-Ara4N) causing a decrease in the net negative charge of lipid A, a constituent moiety of LPS [5–6] and hence resulting in resistance to polymyxin B. The other common method resulting in the modification of lipid A, is the addition of phosphoethanolamine (PEtN) resulting in a decrease in the net negative charge causing a shift towards cationicity with decreased binding affinity for polymyxin B, resulting in eventual resistance to polymyxins [5–6]. The synthesis and transfer of L-Ara4N and PEtN to lipid A resulting in polymyxin resistance is triggered by the activation of PhoP/PhoQ and PmrA/PmrB, the two-component systems (TCS) due to environmental stimuli like drug concentrations, changes in growth conditions or specific mutations occurring within TCS will result in the upregulation of operons that facilitate [6–8].

Most of the mathematical models to date describe the emergence of resistance in response to drug pressure as a mixture of bacterial subpopulations with differing degrees of antibiotic susceptibilities [9–10]. Drug effects depend on the degree of susceptibility of the bacterial subpopulations, and the composition of the total bacterial population exposed to the drug concentration can change over time as bacterial subpopulations which are less susceptible can become dominant. Such models typically assume acquired resistance due to bacterial mutations which determine the initial (prior to drug treatment) partition of the total bacterial population into susceptible subpopulations.

Physiologically structured population (PSP) models were first introduced in ecology to describe the behavior of a population of individual organisms sharing and interacting in a common environment [11]. A key concept of structure as the unique subject characteristics can be extended to cell protein expression, DNA, and RNA content [12–13]. A PSP model describes dynamics of a structure within a bacterium and quantifies its distribution within the bacterial population. The environment can affect both a bacterium and the entire bacterial population. For describing *in vitro* bacteria, the growth media constitutes the environment. In particular, the drug concentration is the most relevant environmental variable determining the dynamics of bacterial population. In the context of Gram-negative bacteria resistance to polymyxin B, a structure of interest will be the expression of LPS on the outer membrane. PSP models are designed to quantify heterogeneity of the bacterial structure and the impact of the environment on the dynamics of bacterial populations.

Our leading hypothesis is that resistance of *Klebsiella pneumoniae* to polymyxin B can be at least partially attributed to a drug-induced selection of a subpopulation due to the heterogeneity of LPS binding in the bacterial population. We apply a PSP model to describe the time-kill kinetics data for two clinical strains with differing susceptibilities to polymyxin B with information regarding the distribution of binding affinities, maximal efficacies, and critical concentrations of polymyxin B necessary for the emergence of resistant bacterial subpopulations. First, we briefly introduce a general framework for PSP models. Next we apply the pharmacological principles of receptor occupancy to introduce binding structures and define the bactericidal effect of the drug. The resulting model is fitted to the experimental time-kill kinetics data and furthermore the parameter estimates were used to simulate the process of selection of resistant subpopulations by exposure to the drug.

## Materials and methods

### *In vitro* static time-kill study

*Klebsiella pneumoniae* Carbapenemase (KPC) producing *Klebsiella pneumoniae* clinical strains BAA1705^™^ (obtained from ATCC^®^) and KP619 (obtained from a patient treated for bacteraemia at the Kingman Regional Medical Center, Kingman, Arizona) were used. MICs (minimum inhibitory concentrations) were determined in triplicate by broth microdilution as per the Clinical and Laboratory Standards Institute guidelines (Clinical and Laboratory Standards Institute. 2014. Performance Standards for Antimicrobial Susceptibility Testing; Twenty-Fourth Informational Supplement. CLSI document M100-S24. Clinical and Laboratory Standards Institute, Wayne, PA).

Mueller-Hinton broth (Becton, Dickinson and Company, Sparks, MD) supplemented with calcium and magnesium (CAMHB; 25.0mg/L Ca^2+^, 12.5mg/L Mg^2+^) was used for susceptibility testing and *in vitro* time-kill models. Stock solutions of polymyxin B (Sigma-Aldrich, St. Louis, MO, Lot. WXBB4470V) were freshly prepared in sterile water prior to each experiment. All drug solutions were filter sterilized using a 0.22μm filter (Fisher Scientific). Static time-kill experiments were performed over 48 h to evaluate pharmacodynamic activity of polymyxin B monotherapy against the two *K*. *pneumoniae* clinical strains. The rate and extent of killing polymyxin B 0.5, 1, 2, 4, 8, and 16 mg/L was assessed. Polymyxin B was added to the logarithmic-phase broth culture prepared prior to each experiment by adding fresh bacterial colonies from overnight growth to pre-warmed CAMHB (37°C) to achieve the desired initial inoculum of ~10^6^ cfu/mL. Serial samples were obtained at 0, 1, 2, 4, 6, 8, 24, 28, 32, and 48 h for bacterial quantification.

### PSP model

PSP models were originally introduced to describe dynamics of population of animals in their habitat [11,14]. However, the theoretical framework applies to any population of individuals living in a specific environment. A PSP model describes the dynamics of a population in terms of the behavior of its constituent individuals. It consists of *i*-state equations:

$$\frac{dx}{dt}=g\left(E,x\right),\;t>0,\;x\in \Omega$$ (1)

where ***x*** is an *n*-dimensional vector of individual structures (*i*-state), *Ω* is the space containing all individual states, and *E* is a vector of environmental variables influencing individual states that is described by *e*-state equations:

$$\frac{dE}{dt}=f\left(E,\psi \left(\text{n}\left(\cdot ,\text{t}\right)\right),\text{t}\right),\text{ t}>0$$ (2)

where *n(****x***, *t)* is the density distribution of the structure ***x*** at time *t* (*p*-state) and Ψ(*n*(∙, *t*)) is a population characteristic (e.g. population size) defined by the *p*-state. For example, by definition of the density function, the number of subjects in the population at time *t* is

$$N\left(t\right)=\int_{\Omega }^{}n\left(x,t\right)dx$$ (3)

*P*-state equations are central for the PSP model:

$$\frac{\partial n\left(x,t\right)}{\partial t}+div\left(g\left(E,x\right)n\left(x,t\right)\right)=\lambda \left(E,\Psi \left(n\left(\cdot ,t\right),x,t\right)\right)-\mu \left(E,\Psi \left(n\left(\cdot ,t\right),x\right)\right)n\left(x,t\right),\;t>0,x\in \Omega$$ (4)

where *λ*(***E***, Ψ(*n*(∙, *t*), ***x***, *t*)) is the density of production rate and *μ*(***E***, Ψ(*n*(∙, *t*), ***x***)) is the mortality rate, for individuals of structure ***x***. The *p*-state Eq (4) uniquely defines the density *n(****x***, *t)* if boundary and initial conditions are specified:

v(x)⋅g(E,x)n(x,t)=α(x,t), t>0, x ϵ ∂Ω (5)

and

$$E\left(0\right)=E_{0},\;n\left(x,0\right)=n_{0}\left(x\right),\;x\;\epsilon \;\Omega$$ (6)

where ***υ***(***x***) is the unit inward-pointing normal vector at ***x*** *ϵ*∂Ω, and *α*(***x***, *t*) and *n*_0_(***x***) are known functions.

#### Binding-structured population models of bacteria *in vitro*

We will consider bacteria growing in the *in vitro* static time-kill model to be exposed to a polymyxin B concentration of *C*. The drug concentration constitutes an environmental variable (*e*-state) that affect the status of each bacterium. Since polymyxin B is stable and the degradation is minimal for the duration of the experiment, we assume that drug concentration in the medium does not change over time:

$$\frac{dC}{dt}=0$$ (7)

Eq (7) is an analog of the e-state Eq (2). Based on the pharmacological principles of drug-receptor theory, the drug effect on each bacterium is determined by the number of receptors bound to the drug (*b*) [15]. The number of bound receptors b depends on the drug concentration in medium C and the number of free receptors per bacterium r, and the number of bacteria N in the culture. Since the time scale for binding and receptor turnover is much smaller as compared to the time scale for the growth of bacteria we assume that the binding is at dynamic equilibrium. Then the bound receptor on each cell can be calculated as

$$b=\frac{r_{tot}C}{K_{D}\;+\;C}$$ (8)

where *K*_*D*_ is the equilibrium disassociation constant and *r*_*tot*_ describes the total number of receptors per bacterium that is assumed to be constant:

$$r_{tot}=r+b$$ (9)

Consequently, under equilibrium assumptions, we get $\;r=\frac{r_{tot}K_{D}}{K_{D}+C}$, hence the number of free and bound receptors on a bacterial cell is determined by *r*_*tot*_ and *K*_*D*_ specific for that cell. In this context, a relevant structure determining the status of each bacterium is a vector (*r*_*tot*_, *K*_*D*_).

In this section we have ***x*** = (*r*_*tot*_, *K*_*D*_) and ***E*** = *C*. Since the bacterial cell binding parameters are constant, the i-state Eq (1) becomes:

$$\frac{d}{dt}\left(r_{tot},K_{D}\right)=\left(0,0\right)=g\left(C,r_{tot},K_{D}\right)$$ (10)

Under these assumptions the p-state Eq (4) describing the receptor density n(*r*_*tot*_, *K*_*D*_, *t*) simplifies to

$$\frac{\partial n\left(r_{tot},K_{D},t\right)}{\partial t}=\lambda \left(C,\;N,r_{tot},K_{D},t\right)-\mu \left(C,\;N,r_{tot},K_{D}\right)n\left(r_{tot},K_{D},t\right),\;r_{tot}>0,\;K_{D}>0,\;t>0$$ (11)

with the initial condition

$$n\left(r_{tot},K_{D},0\right)=n_{0}\left(r_{tot},K_{D}\right),\;r_{tot}>0,\;K_{D}>0$$ (12)

#### Cytotoxic effect

The mortality rate *μ*(*C*, *N*, *r*_*tot*_, *K*_*D*_) is assumed to be independent of the bacteria population size and is stimulated by the drug:

$$\mu \left(C,N,r_{tot},K_{D}\right)=\mu_{0}S\left(b\right)$$ (13)

where *μ*_0_ is the first-order death rate constant and *S*(*b*) is the stimulatory function. We assume that the drug effects are characterized as power functions of *b*

$$S\left(b\right)=1+\kappa b^{\gamma }$$ (14)

where *γ* is the power coefficient describing the steepness of the stimulatory curve and *κ* is the coefficient.

#### Modeling bacteria growth

To accurately describe the dynamics of bacteria growth *in vitro* we will adopt existing models [16]. The bacterial density growth rate *λ*(*C*, *N*, *r*_*tot*_, *K*_*D*_) is assumed to slow down with an increasing bacteria population size:

$$\lambda \left(C,\;N,r_{tot},K_{D},t\right)=\frac{VG_{max}}{N_{m}+N}n\left(r_{tot},K_{D},t\right)$$ (15)

where *VG*_*max*_ is a maximal velocity of bacterial growth, and *N*_*m*_ denotes the bacterial count that results in 50% of the maximal rate of growth. If the size of the bacteria population is much less than *N*_*m*_ i.e.

$$N\ll N_{m}$$ (16)

then the growth rate becomes linear:

$$\lambda \left(C,N,r_{tot},K_{D},t\right)=\lambda_{0}n\left(r_{tot},K_{D},t\right)$$ (17)

where *λ*_0_ = *VG*_*max*_/*N*_*m*_. Integrating p-state Eq (11) one can arrive at a solution:

$$n\left(r_{tot},K_{D},t\right)=n_{0}\left(r_{tot},K_{D}\right)exp\left((\lambda_{0}-\mu_{0}S\left(b\right))t\right)$$ (18)

Where bound receptor *b* is determined by Eq (8). In the case of point distribution of *r*_*tot*_ ≡ *r*_*tot*0_:

$$n\left(K_{D},t\right)=n_{0}\left(K_{D}\right)exp\left((\lambda_{0}-\mu_{0}S\left(b\right))t\right)$$ (19)

where

$$n_{0}\left(K_{D}\right)=N_{0}\frac{\beta }{\alpha }{\left(\frac{K_{D}}{\alpha }\right)}^{\beta -1}exp\left(-{\left(\frac{K_{D}}{\alpha }\right)}^{\beta }\right)$$ (20)

#### Cell count response

The bacterial cell count in the population can be calculated by integration of the density *n*(*r*_*tot*_, *K*_*D*_, *t*) as shown in Eq (3). Integrating of Eq (18) over *r*_*tot*_ and *K*_*D*_ yields:

$$N\left(t\right)=\int_{0}^{\infty }\int_{0}^{\infty }n_{0}\left(r_{tot},K_{D}\right)exp\left((\lambda_{0}-\mu_{0}S\left(b\right))t\right)d\;r_{tot}d\;K_{D}$$ (21)

In the absence of the drug (*S*(*b*) ≡ 1) the bacteria population grows exponentially:

$$N\left(t\right)=N_{0}exp\left((\lambda_{0}-\mu_{0})t\right)$$ (22)

with the half-life

$$t_{1/2}=\frac{\text{ln}\left(2\right)}{\lambda_{0}-\mu_{0}}$$ (23)

In the case of point distribution of *r*_*tot*_ ≡ *r*_*tot*0_:

$$N\left(t\right)=\int_{0}^{\infty }n_{0}\left(K_{D}\right)exp\left((\lambda_{0}-\mu_{0}S\left(\frac{r_{tot0}C}{K_{D}+C}\right))t\right)d\;K_{D}$$ (24)

whereas all cells have the same affinity *K*_*D*_ ≡ *K*_*D*0_:

$$N\left(t\right)=\int_{0}^{\infty }n_{0}\left(r_{tot}\right)exp\left((\lambda_{0}-\mu_{0}S\left(\frac{r_{tot}C}{K_{D0}+C}\right))t\right)d\;r_{tot}$$ (25)

#### Initial distribution of *r*_*tot*_ and *K*_*D*_

The last part of the model to be defined is the initial density of *r*_*tot*_ and *K*_*D*_. Anticipating limited information from *in vitro* bacteria growth data about the expression of receptors and bacteria affinities to drug, a parsimonious approach was taken to describe *n*_0_(*r*_*tot*_, *K*_*D*_). A natural assumption is independence of *r*_*tot*_ and *K*_*D*_ distributions resulting in the product:

$$n_{0}\left(r_{tot},K_{D}\right)=N_{0}p_{0}\left(r_{tot}\right)q_{0}\left(K_{D}\right)$$ (26)

where *p*_0_(*r*_*tot*_) and *q*_0_(*K*_*D*_) are probability density functions (pdfs) for *r*_*tot*_ and *K*_*D*_ distributions, respectively. Two type of pdfs were tested to account for extreme cases: Weibull

$$p\left(x\right)=\frac{\beta }{\alpha }{\left(\frac{x}{\alpha }\right)}^{\beta -1}exp\left(-{\left(\frac{x}{\alpha }\right)}^{\beta }\right)$$ (27)

where *α* is the scale parameter and *β* is the shape parameter, and point distribution

$$p\left(x\right)=\delta \left(x-x_{0}\right)$$ (28)

where *x*_0_ is the center of the distribution. The point distribution assumes all cells have the same structure value (*r*_*tot*_ or *K*_*D*_).

### Data analysis

The model parameters were estimated using the SAEM algorithm implemented in Monolix 4.3.3 (Lixoft). Simulations were prformed in MATLAB (R2013a, MathWorks). The numerical evaluation of the integrals was done by the MATLAB function *integral* available to Monolix.

## Results

### Mechanisms of resistance for bacterial population with unrestricted growth

The binding-structured population model offers an explanation for a mechanism of resistance of bacteria in response to treatment with a bactericidal antibiotic based on the balance between production and elimination rates that can vary between bacteria as a consequence of bacteria specific binding of the drug. For the unrestricted growth of bacteria, the time course of the density function *n*(*r*_*tot*_, *K*_*D*_, *t*) is described by the p-state equation:

$$\frac{\partial n\left(r_{tot},K_{D},t\right)}{\partial t}=\left(\lambda_{0}-\mu_{0}S\left(b\right)\right)n\left(r_{tot},K_{D},t\right)$$ (29)

which can be solved to yield Eq (11). Consequently, the curve

$$\lambda_{0}-\mu_{0}S\left(b\right)=0$$ (30)

divides the plane (*r*_*tot*_, *K*_*D*_) into two regions. Bacteria in the region for which the left hand side of Eq (30) is negative will exponentially die, whereas bacteria in the complementary region will grow exponentially. This is a resistance mechanism where the drug selects bacteria in the initial bacterial population *n*_0_(*r*_*tot*_, *K*_*D*_, *t*) for death or survival depending on their binding characteristics [2]. Bacteria expressing large number of receptors or with strong binding affinity are likely to die, while the bacteria expressing few receptors or with weak binding affinity for the drug will survival forming a new more resistant bacterial population growing exponentially in time. The time for the emergence of this new more resistant bacterial population depends on the proportion of the initial distribution contained in the growing region of the (*r*_*tot*_, *K*_*D*_) plane. According to Eq (30) there exists a critical number of bound receptors per bacterium *b*_*crit*_ such that a subpopulation of bacteria expressing bound receptors *b* < *b*_*crit*_ will continue to grow. This happens as the elimination rate of bacteria is determined by the hazard of death which is less than the production rate of bacteria determined by *λ*_0_. While the subpopulation of bacteria with bound receptors *b* > *b*_*crit*_ diminishes as the hazard of death is greater than the production rate of bacteria.

$$\lambda_{0}=\mu_{0}S\left(b_{crit}\right)$$ (31)

**Fig 1:**
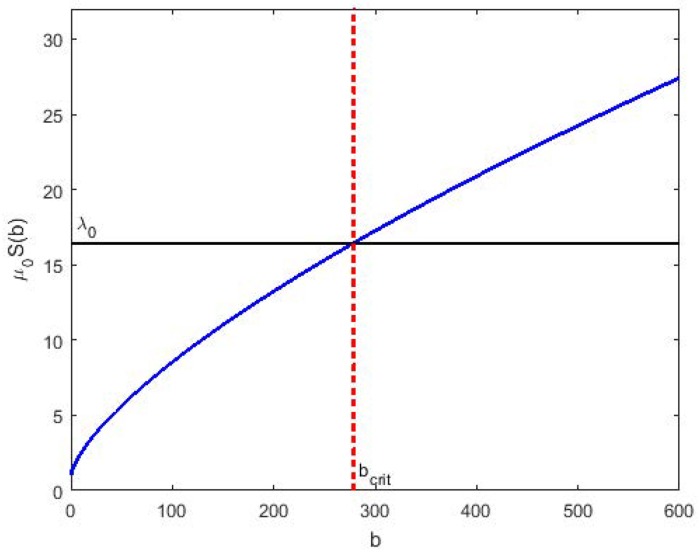
The mortality rate as a function of the number bound receptors per cell. The hazard of cell removal *μ*_0_*S*(*b*) increases as an effect of drug action *S*(*b*) starting from the baseline value *μ*_0_. The horizontal line marks the first-order production rate constant for cell population *λ*_0_. The interception of two lines determines the critical value of bound receptors *b*_*crit*_. Cells with *b < b*_*crit*_ will continue to grow whereas cells *b*_*crit*_ < *b* are destin to die.

Fig 1 illustrates the balance between the hazard of bacterial death and the first-order production that defines the critical number of bound receptors available in the population for the drug to exert its effect.

To demonstrate this mechanism we will use an example of bacteria that differ only in terms of their binding affinity *K*_*D*_ while expressing the same number of receptors *r*_*tot*0_. Then a two dimensional density *n*(*r*_*tot*_, *K*_*D*_, *t*) can be reduced to a one dimensional density *n*(*K*_*D*_, *t*). Since the number of bound receptors is uniquely determined by bacteria binding characteristics Eq (8), there exists a critical equilibrium dissociation constant *K*_*Dcrit*_ such that a subpopulation of bacteria with *K*_*D*_ > *K*_*Dcrit*_ continue to grow whereas a subpopulation of bacteria *K*_*D*_ < *K*_*Dcrit*_ are decreasing:

$$K_{Dcrit}=C\left({\left(\frac{\mu_{0}\kappa r_{tot0}{}^{\gamma }}{\lambda_{0}-\mu_{0}}\right)}^{1/\gamma }-1\right)$$ (32)

Eq (32) implies that necessary and sufficient condition for existence of bacteria as *K*_*Dcrit*_ is

$$\kappa r_{tot0}{}^{\gamma }>\frac{\lambda_{0}}{\mu_{0}}-1>0$$ (33)

In particular, Eq (33) implies that if bacteria express a low number of receptors, then *K*_*Dcrit*_ may not exist and the original bacteria population will continue to grow exponentially, entirely resistant to drug effect.

### Resistance of Gram-negative bacteria to polymyxin B

**Fig 2:**
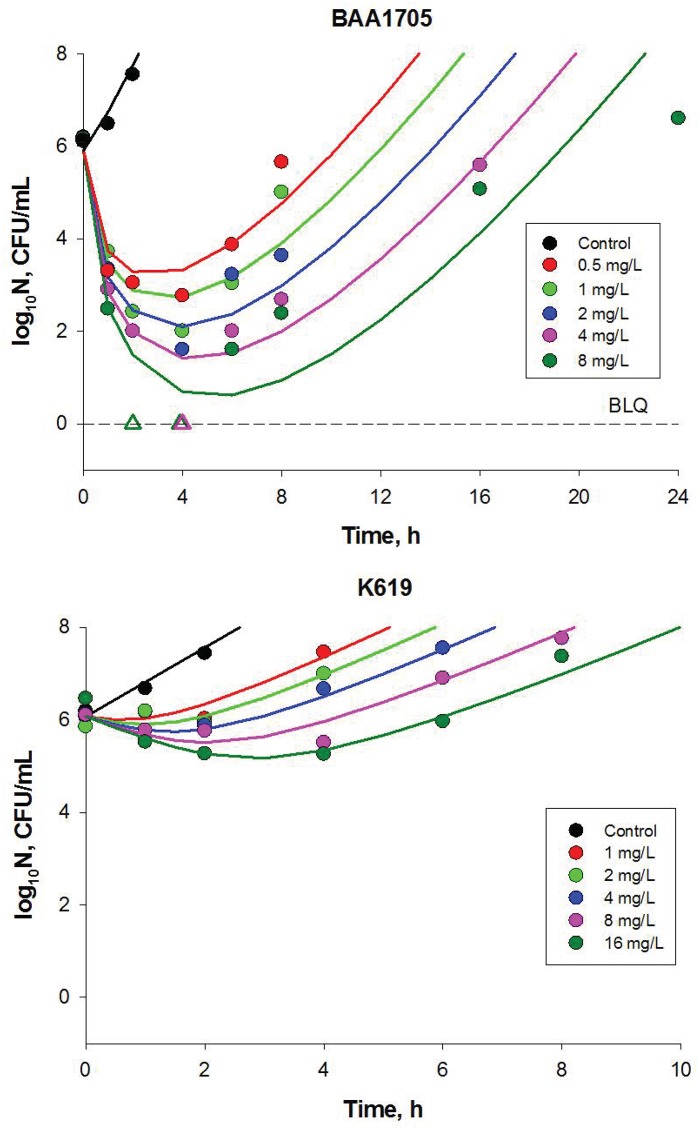
Time-kill data for BAA1709 (upper panel) and KP619 (lower panel) strain. Symbols represent the measurements and lines are model fitted curves. The dashed line marks the limit of quantification of 1 cfu/mL. The triangle symbols are measurements below limit of quantification.

**Fig 3:**
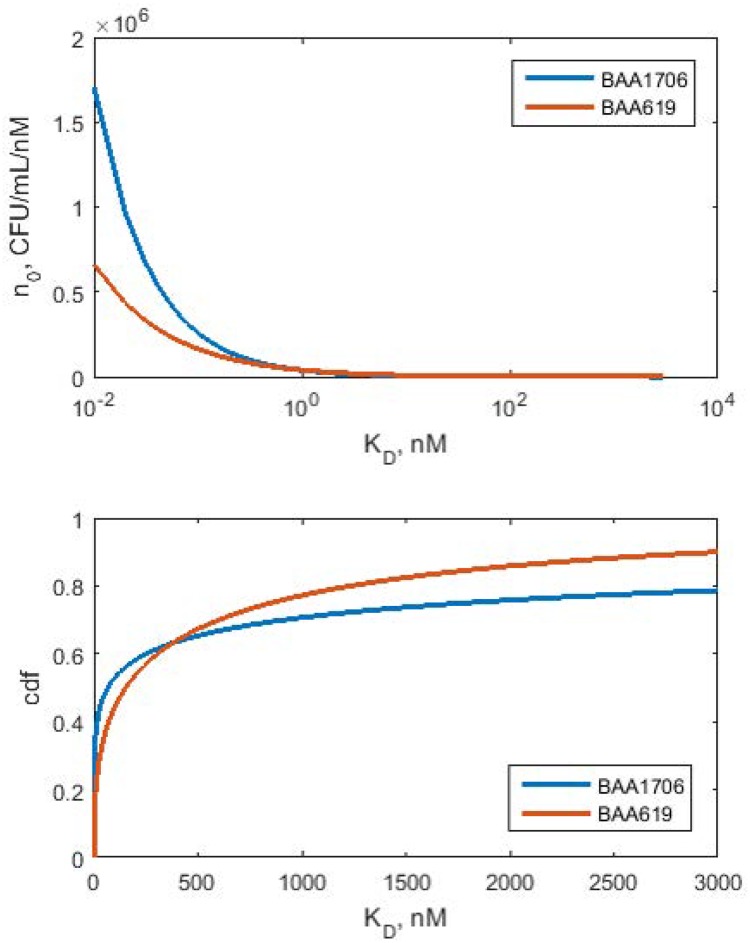
Initial *K*_*D*_ density distributions (upper panel) and cumulative distribution functions (lower panel) for BAA1705 and KP619 strains. Estimated parameter values from Table 1 were used to simulate *n*_0_(*K*_*D*_) and corresponding cdfs based on Eq (20).

Two clinical strains of KPC producing *Klebsiella pneumoniae* with differing susceptibilities to polymyxin B were grown in Mueller Hinton broth, growth media containing escalating polymyxin B concentrations over 48 h. For the susceptible strain BAA1705 the minimum inhibitory concentration (MIC) for polymyxin B is 0.5 mg/L whereas for the more resistant strain KP619 MIC for polymyxin B is 64 mg/L. Time-kill kinetics for both strains are shown in Fig 2. In time-kill studies, polymyxin B was bactericidal in a concentration-dependent manner Fig 2. All polymyxin B concentrations evaluated resulted in a >3 log_10_ reduction against BAA1705 by 2–4 h and all strains regrew until they were similar to growth control or until they reached the threshold. Against KP619, a less than 1-log_10_ reduction was seen at concentrations greater than 4 mg/L that was not sustained beyond 4 h and regrowth was seen at all evaluated concentrations. To ensure exponential growth the control data were used to determine a threshold of 10^8^ cfu/mL to censor the data for all treatment groups when the size of the bacteria population increases above the threshold. The rate (or initial slope of time-kill curves) was greater and extent of killing by polymyxin B for BAA1705 was more extensive as compared to that for KP619, consistent with their reported polymyxin B susceptibilities. The time-kill data imply the emergence of bacteria resistant to polymyxin beyond 4–6h of exposure to polymyxin B. According to the assumption of heterogeneity in polymyxin B affinities of the receptors expressed by the bacteria populations evaluated here, the time-kill kinetics data were fitted by the model Eq (23). The resulting fits are shown in Fig 2 and parameter estimates and their 95% CIs are presented in Table 1. Two model parameters could not be estimated based on the available data and hence they were fixed. The bacterial natural death rate constant (baseline hazard), *μ*_0_ was set to the value 0.3 day^-1^ reported elsewhere [17]. The scale parameter *α*, for the initial distribution was set at the value of 380 nM, the equilibrium dissociation constant for polymyxin B binding to LPS [18]. The estimated shape factors *β* were < 1 implying right-skewed distributions with singularity at *K*_*D*_ = 0. Since 95% CIs corresponding to BAA1705 and KP619 strains are disjoint, the K_D_ distributions are significantly different which is confirmed by Fig 3. The dimensional analysis revealed that parameters *κ* and *r*_*tot0*_ can be estimated only as a part of the term *vr*_*tot0*_^*γ*^. The latter can be interpreted as the drug effect corresponding to 100% receptor occupancy, a maximal stimulation for the given number of receptors per bacterium.

**Table 1:** Parameter estimates of model Eq (24) obtained by fitting the time-kill data for BAA1705 and KP619 strains shown in Fig 2.

Parameter	Estimate (%RSE) BAA1705	95% CI BAA1705	Estimate (%RSE) KP619	95% CI KP619
*λ*_*0*_, day^-1^	3.27 (22)	[1.80,4.74]	2.02 (9)	[1.63;2.41]
*μ*_*0*_, day^-1^	0.3*		0.3*	
*κr*_*tot0*_^*γ*^	61.2 (35)	[18.4,104.0]	10.1 (8)	[8.34,11.6]
*γ*	0.186 (32)	[0.0657,0.306]	0.831 (29)	[0.335,1.33]
log_10_*N*_*0*,_ cfu/mL	5.92 (5)	[5.35,6.49]	6.07 (1)	[5.93,6.21]
*α*, nM	380*		380*	
*β*	0.211 (13)	[0.154,0.268]	0.404 (9)	[0.334,0.474]

%RSE stands for percent relative standard error of the estimate.

*—parameter was fixed.

### Distribution of affinities to polymyxin B

The model structure and parameter estimates allows one to make inferences about the initial distribution of *K*_*D*_ among the bacteria and the shape of the hazard function for escalating drug concentrations. Fig 3 reveals the inferred *K*_*D*_ density distributions after initiating treatment with polymyxin B against both clinical isolates evaluated. The estimated shape factors *β* were < 1 implying right-skewed distributions with singularity at *K*_*D*_ = 0. The cumulative distribution functions yield the following quartiles Q_1_ = 1.06 nM, Q_2_ = 67 nM, and Q_3_ = 1788 nM (BAA1705), and Q_1_ = 17.4 nM, Q_2_ = 154 nM, and Q_3_ = 853 nM (K619). This implies that more than half of both bacterial isolates have stronger affinity for polymyxin B than reported 380 nM. Also, about quarter of the bacteria bind to polymyxin B in the micro molar range. As expected, BAA1705 isolate has more polymyxin B susceptible bacteria with *K*_*D*_ < 100 nM as compared to the more resistant KP619 isolate (53% vs. 44%).

### Cytotoxic effect of polymyxin B

**Fig 4:**
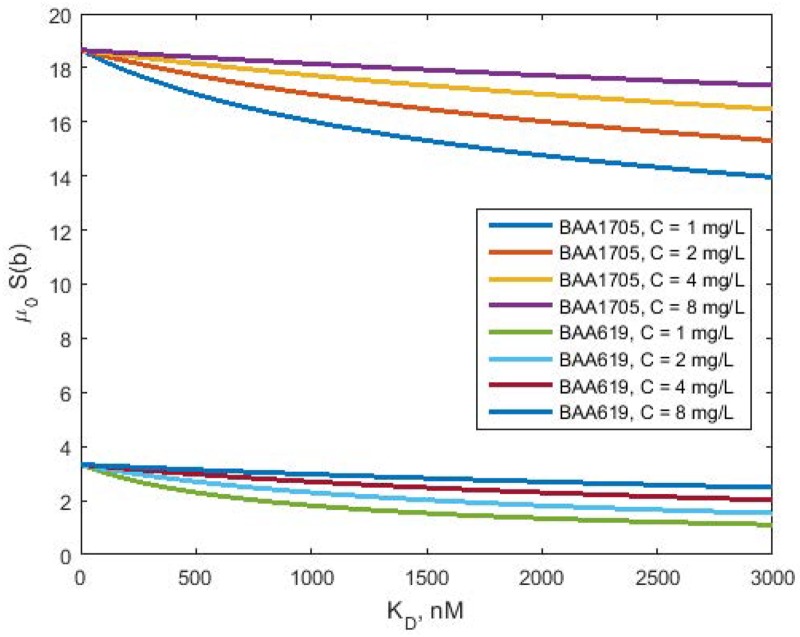
Hazard as functions of *K*_*D*_ for BAA1709 and KP619 strains. The curves *μ*_0_*S*(*b*) vs. *K*_*D*_ were simulated using Eq (34) for indicated drug concentrations. Parameter values used for simulations are presented in Table 1.

The estimates of *γ* and *κr*_*tot0*_^*γ*^ are sufficient to generate plots of the hazard *μ*_0_*S*(*b*) as functions *K*_*D*_ shown in Fig 4:

$$\mu_{0}S\left(b\right)=\mu_{0}\left(1+\kappa r_{tot0}{}^{\gamma }{\left(\frac{C}{K_{D}+C}\right)}^{\gamma }\right)$$ (34)

At *K*_*D*_ = 0 nM the maximum hazards of bacteria death were 18.7 day^-1^ and 3.3 day^-1^ for BAA1705 and KP619, respectively. The hazards decreased with increasing *K*_*D*_ and were dependent on the polymyxin B concentration. Higher polymyxin B concentrations resulted in higher hazards of death. The sensitivity of the hazard to drug concentration was higher for BAA1705 compared to that for KP619, consistent with values of *γ* parameter as the power of the receptor occupancy *C*/(*K*_*D*_ + *C*).

### Selection of resistant cell population

**Fig 5:**
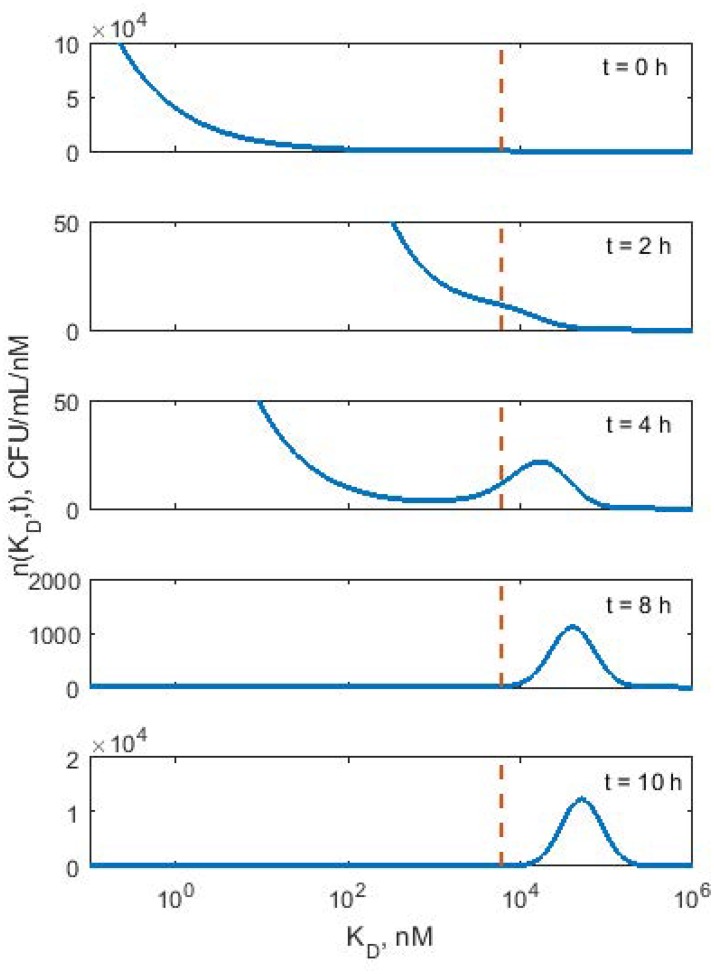
Simulated density distributions of *K*_*D*_ at various times for KP619 bacteria population exposed to polymyxin B concentration of *C* = 8 mg/L. Si The vertical line indicates *K*_*Dcrit*_ = 6003 nM. The ranges of y axes were adjusted to best illustrate the shape of distribution. At *t* = 0 the distribution is equal to the initial density *n*_0_(*K*_*D*_) described by the Weibull distribution Eq (16). As time progresses the density of cells with *K*_*D*_ < *K*_*Dcrit*_ vanishes whereas cells with *K*_*D*_ > *K*_*Dcrit*_ form a new population that eventually grows exponentially. The density distribution at *K*_*D*_ = *K*_*Dcrit*_ remains constant.

The affinity of bacteria to polymyxin B, *K*_*Dcrit*_ determines its fate, when it is exposed to the cytotoxic effect of drug. Bacteria with *K*_*D*_ > *K*_*Dcrit*_ will grow, whereas bacteria with *K*_*D*_ < *K*_*Dcrit*_ are destined to be killed by the drug. This is the basic mechanism of selection for resistant bacteria based on their affinity to drug. Fig 5 shows the density *n*(*K*_*D*_, *t*) at various times for KP619 exposed to polymyxin B concentration of C = 8 mg/L to illustrate the emergence of resistant bacteria subpopulation with *K*_*D*_ > *K*_*Dcrit*_ = 6003 nM. At time *t* = 0 h, only 4.7% of *N*(0) = 1.2 x10^6^ cfu/mL bacteria had *K*_*D*_ > *K*_*Dcrit*_. At *t* = 2 h, this fraction increased to 52.7% of *N*(2) = 3.2 x10^5^ cfu/mL, and by *t* = 4 h almost the entire bacteria population 95.5% of *N*(4) = 9.1x10^5^ cfu/mL became resistant, and the process of growth dominated the killing. At *t* = 8 h more than 99.9% of *N*(8) = 7.8 x10^7^ cfu/mL bacteria had *K*_*D*_ > *K*_*Dcrit*_, and consequently the population size increased exponentially with time. This suggests that bacteria with weak binding affinity to the drug become resistant, while bacteria with strong binding affinity to the drug are eliminated. Another observation is that the drug concentration, *C* is also a determinant of the emergence of resistant bacterial population. The time scale for emergence of the resistant population depends on the percentile of the initial distribution cut off by *K*_*Dcrit*_. The smaller the tail is, the longer it takes for the resistant bacteria population to emerge. Lastly, over time as the total bacteria population becomes resistant, the *K*_*D*_ values shifts to higher values or *K*_*D*_. values tend to increase. The mode at *t* = 8 h was 40,306 nM whereas at *t* = 10 h it was 51,042 nM. The process of selection resistant population for BAA1705 strain was qualitatively similar to that for KP619 strain.

## Discussion

Existing *in vitro* models of infection account for resistance in response to drug pressure by describing the total bacterial population by considering a discrete number of bacterial subpopulations with varying degrees of susceptibility to the antibiotic(s) being evaluated (see for example [9]). The approach presented here fundamentally differs from assuming a priori existence of several bacterial subpopulations as opposed to one that evolves in time upon exposure to drug resulting in the emergence of a resistant population eventually. The discrete susceptibilities of bacteria to drug is explained by a continuous distribution of drug binding characteristics within the bacterial population. A resistant population is determined by the balance between the growth and death processes altered by the drug. As such, the distribution will change in time where the concentration of the drug is the forcing function.

Since resistance emerges at low bacteria counts, our model of bacteria growth was limited to a first-order process with drug stimulating bacterial death. This simplification was intentional to arrive at a mathematically simpler model allowing for explicit criteria determining the critical binding characteristics necessary to define a resistant bacteria population. Consequently, the model can be applied to the time-kill kinetics data only within the exponential growth phase. Given the two characteristics of receptor density, *r*_*tot*_ and affinity for drug, *K*_*D*_, the model was further simplified by assuming that these characteristics do not vary between bacteria. This was necessary since the available data did not support identifiability of these parameters for a two-dimensional distribution. The model allowed for a rather flexible initial distribution of binding characteristics (Weibull function), and a relatively general model of the effect of the drug on the hazard of cell death (power function of bound receptors per cell).

The model was qualified against time-kill kinetics data for two isolates of KPC producing *Klebsiella Pneumoniae* with differing susceptibilities to polymyxin B with the assumption of the same target expression within the bacterial population. Parameter estimates revealed similar qualitative characteristics but difference in numerical values. The initial distributions of *K*_*D*_ for both strains were centered near the vicinity of *K*_*D*_ = 0 with right tails extending to infinity with about 25% of bacteria in the micro-molar range. The drug effect on the death hazard differed in its maximal value being 6-fold stronger for the more susceptible bacteria strain, BAA1705. Also, the sensitivity of the more susceptible bacteria strain to drug concentration was higher.

The selection of functions describing initial distributions of target within the bacteria population and drug effects on the death hazard was parsimonious. While the Weibull probability density function guaranteed sufficient flexibility, a scale parameter could not be identified. The power function Eq (20) for describing the drug effect was a reduced form of the sigmoidal E_max_ model recommended by the operational model of agonism [19]. Such an extension would allow for maximal saturation of the bacteria killing when the number of bound receptors per bacterium is high. This feature might be of importance for selection resistance mechanisms due to the distribution of *r*_*tot*_ within the population which have not been explored in this report. This part of our modeling approach indicates limitation of time-kill kinetics data in order to make inferences about mechanisms of resistance and drug pharmacodynamics. Additional information about time courses of receptor expression on bacteria exposed to drug effect is warranted.

The mathematical framework of structured populations was applied to describe the time courses of the *K*_*D*_ distributions in its simplest form. Constant drug concentrations eliminated the need of modeling the environment (pharmacokinetic model was absent). However, this assumption might be violated if the size of bacteria population is large enough to clear drug from the medium. The assumption of binding equilibrium between drug and receptors yielded a trivial bacteria level model of the receptor turnover and allowed for introducing *K*_*D*_ as a structure. This limited the mechanism of resistance to a passive selection of bacteria by the drug based on the distribution of *r*_*tot*_ and *K*_*D*_. A more dynamic model (i-state) would allow for description of additional mechanisms of resistance such as down-regulation of the receptor by the bacteria in response to environmental triggers.

In summary, we applied a theory of physiologically structured populations to develop a mathematical model of bacteria resistance to antibiotics *in vitro* that is based on a heterogeneous distribution of receptors and affinities among cells. This model can account for a mechanism of resistance due to selection of bacteria with favorable binding characteristics given particular drug concentration. The model is limited to exponential growth of bacteria with drug stimulating bacterial killing. The resistant population is determined by the balance between growth rate and hazard of cell death. Two bacterial isolates with different susceptibilities to polymyxin B were used for model qualification assuming similar receptor expression on all bacteria. Estimates of shape parameters for distributions of dissociation equilibrium constants yielded unimodal distributions with the modes at 0 nM and the right tails containing approximately 25% of the bacteria. The maximal efficacy of polymyxin B was about 6-fold higher for the more susceptible strain than for less susceptible one. Finally, we observed that the time-kill experimental *in vitro* data contained limited information about the mechanisms of resistance and drug pharmacodynamics, implying that additional experimental techniques using dynamic *in vitro* models of infection might provide data that would allow for less parsimonious models than the one presented here that can be used for analysis.

## Appendix

### Derivation of Eq (18)

Despite of presence of many variables, the *p*-state Eq (11) is a linear ODE with respect to time *t* that can be solved by the integrating factor method. Under the simplifying conditions Eqs (13) and (17), the *p*-state Eq (11) becomes Eq (29). Multiply both sides of Eq (19) by the integrating factor *exp*((−*λ*_0_ + *μ*_0_*S*(*b*))*t*) to arrive at

$$\frac{\partial }{\partial t}\left(n\left(r_{tot},K_{D},t\right)exp\left((-\lambda_{0}+\mu_{0}S\left(b\right))t\right)\right)=0$$ (35)

Hence, the initial condition Eq (12) implies

$$n\left(r_{tot},K_{D},t\right)exp\left((-\lambda_{0}+\mu_{0}S\left(b\right))t\right)=n_{0}\left(r_{tot},K_{D}\right)$$ (36)

and Eq (18) follows.

## Supporting information

S1 Data This is the compressed data file containing the *in vitro* data for both bacterial strains used to develop the model. (7Z)
